# Factors Associated With Functional Limitations in Daily Living Among Older Adults in Korea: A Cross-Sectional Study

**DOI:** 10.3389/ijph.2022.1605155

**Published:** 2022-12-09

**Authors:** Van Cuong Nguyen, SeolHawa Moon, Eunmi Oh, Gwi-Ryung Son Hong

**Affiliations:** ^1^ College of Nursing, Hanyang University, Seoul, South Korea; ^2^ Research Institute of Nursing Science, Hanyang University, Seoul, South Korea

**Keywords:** older adults, demographics, activities of daily living, functional limitations, instrumental activities of daily living

## Abstract

**Objective:** This paper assesses the relationship between demographics, health parameters, and functional limitations among older adults in Korea, including limitations in activities of daily living (ADLs) and instrumental activities of daily living (IADLs).

**Methods:** We analyzed data from the Korean Longitudinal Study of Aging survey in 2020 and included only participants aged 65 and older. Multinomial logistic regression models were conducted to evaluate the factors that predicted functional limitations.

**Results:** The prevalence of at least one ADL and IADL limitations were 6.14% (severe 1.94% and moderate 4.20%) and 15.49% (severe 3.11% and moderate 12.38%), respectively. People aged 85 and older had high rates of severe disability with 7.37% for ADLs and 12.06% for IADLs. High rates also occurred among people with low education, underweight, physical inactivity, depression, and three or more chronic diseases.

**Conclusion:** Factors associated with functional limitations were age, educational status, body mass index, physical activity, depression, and chronic diseases. To prevent and improve functional limitations in the older populations, active and applicable interventions should be considered for modifiable factors such as physical activity, depression, and abnormal weight.

## Introduction

The concept of aging in place, living independently or with some assistance from the community, has been well-acknowledged and promoted by the Korean government for people aged 65 years and older (older adults), including older adults with dementia [1], and it has been popular among that population [2]. However, older adults often experience physiological changes, such as declines in physical, cognitive, and sensory functioning, associated with the aging process [3].

The most common indicators of functional limitations in older adults are changes in the ability to perform activities of daily living (ADLs) and instrumental activities of daily living (IADLs) [4]. ADLs are the basic living skills required to independently care for oneself, such as eating, bathing, and personal mobility. IADLs are more complex activities such as money management and use of transportation [4, 5]. Globally, the prevalence of functional limitations has increased significantly in recent years and burdened the healthcare system. As mentioned in recent studies, around 22% of older adults with dementia in India had difficulty with at least one ADL, and up to 48% had difficulty with at least one IADL [6]. Those proportions among older adults in southeastern Poland were 17% and 36%, respectively [7], they were 16.6% and 40.9% in India [6], and were 5.6% and 12.0% in Korea, respectively [8]. Such functional decline was a strong predictor of institutionalization, which leads to an increase in medical costs [9–11].

Several studies found that the progression of disability in older adults differed by sociodemographic and health-related parameters such as age, sex, education status, living arrangement, physical activity, and body mass index (BMI) [12–16]. The number of chronic diseases, which was considered the primary measure of disease severity [17], was an especially significant factor in ADL/IADL disabilities [4]. Older adults with severe or moderate functional decline tended to remain in those functional decline groups instead of dynamically improving or worsening in physical function [4, 16]. Nevertheless, studies on older adults in Korea only investigated the disability-related factors of ADL and IADL [18–20], but little is known about the factors related to the severity of functional disability, separating by both mild and severe disability. As the severity of functional limitation increases, the rate of disability usually increases [6]. Thus, it must be meaningful to identify the related factors of both moderate and severe disability. To minimize the occurrence of physical disability, which is the cause of a social burden, it is necessary to identify the related factors according to the severity of functional limitations.

Our purposes in this study are to show the prevalence of ADL/IADL disability levels the distribution of ADL/IADL disability level according to age group by sex, and to determine the relating factors of the ADL/IADL disability levels according to demographics and health parameters in older Korean adults.

## Methods

### Data and Participants

**FIGURE 1:**
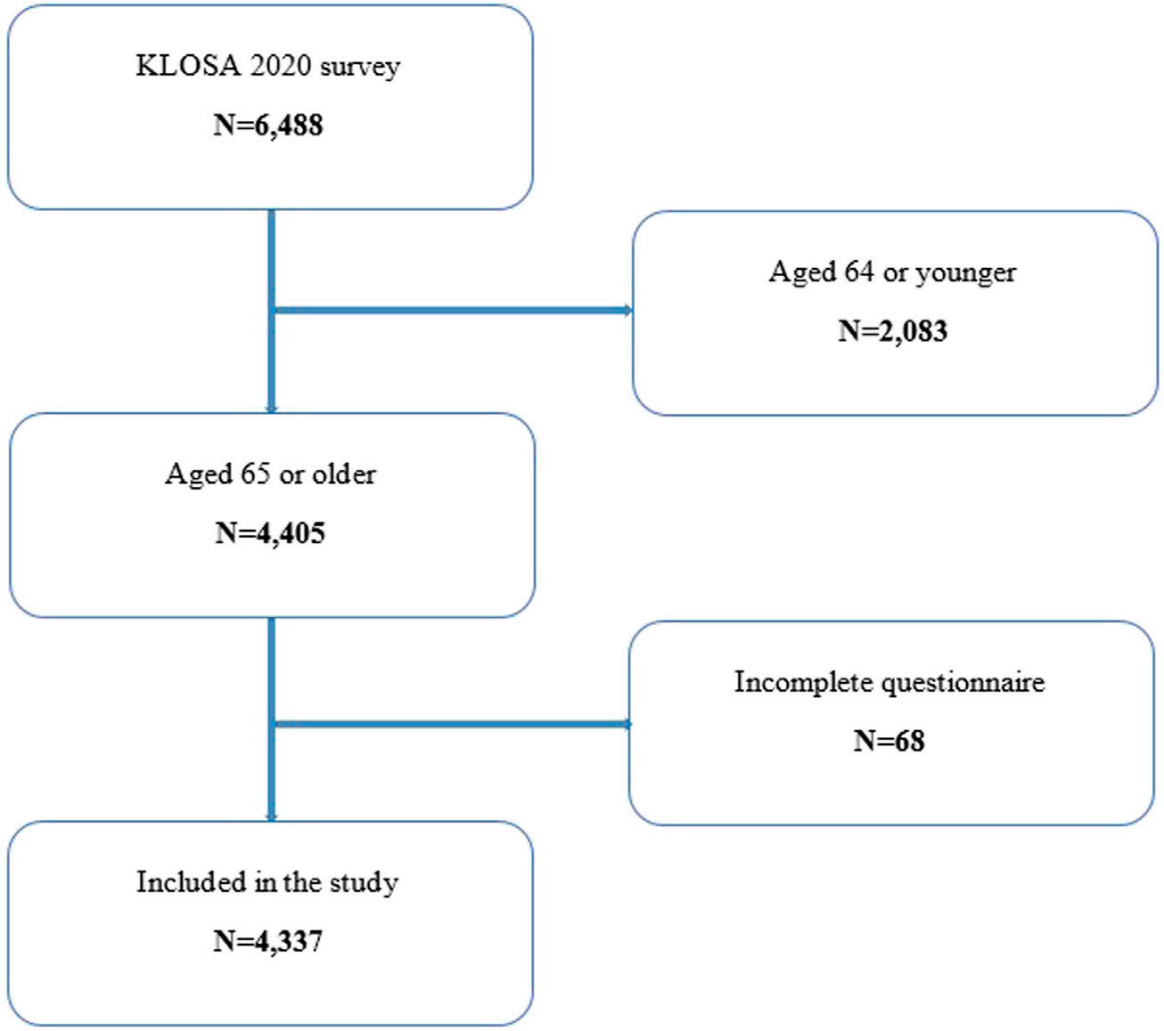
Flow diagram of the study population (the Korean Longitudinal Study of Aging Survey, South Korea, 2020).

In this study, the data from the Korean Longitudinal Study of Aging survey in 2020 (KLOSA 2020) [21] were applied. The survey targeted community-dwelling adults aged 45 and older and covered eight topics affecting adults’ economic and social activities: demographics, family, health, employment, income, assets, subjective expectations, and life satisfaction. Detailed information about this survey is available on the survey organization’s website [21]. We analyzed 4,337 older adults (aged 65 and older) who completed the survey. Figure 1 shows the participant selection and drop-out process.

### Functional Limitations

Following the KLOSA 2020 survey, the ADL scale considered seven indicators: 1) dressing, 2) washing face, hair, and brushing teeth, 3) bathing or showering, 4) eating food that has been prepared, 5) getting in/out of bed, 6) toileting, and 7) managing bladder/bowel [21]. For each indicator, participants were required to respond as independent (score: 0) or dependent (score: 1). An ADL index was then calculated by the sum of scores from seven indicators. In this study, to show that the influence of demographics and health parameters on functional limitation is different, depending on its severity, an ADL disability was defined with three levels of designated severity: “none,” “moderate,” and “severe,” corresponding to participants scoring 0, 1–6, and 7 on the ADL index, respectively.

The IADL scale contained ten instrumental activities: 1) grooming, 2) household chores, 3) preparing meals, 4) laundry, 5) going out a short distance, 6) using public transportation, 7) shopping, 8) managing money, 9) making or taking a phone call, and 10) taking medicine at the right dose and time. The IADL index had a total of 10 possible scores, calculated based on the sum of scores from ten indicators. With the same meaning as ADL disability, an IADL disability was also defined with three levels: “none,” “moderate,” and “severe,” corresponding to participants scoring 0, 1-9, and 10 on the IADL index, respectively. This classification of ADL disability and IADL disability into different levels is similar to that of previous studies [6].

### Covariates

The covariates were taken from the KLOSA 2020 data and included demographic factors of sex (male and female), age (65–74, 75–84, and 85 years or older), living arrangement (with relatives and living alone), and educational status (elementary school or lower, middle school, high school, and college or higher); and health parameters of BMI (underweight, <18.5 kg/m^2^; normal, 18.5–23 kg/m^2^; overweight, 23–25 kg/m^2^; and obesity, >25 kg/m^2^, based on the Asia-Pacific classification of BMI [22]), physical activity (yes and no, in response to answering the question: “How often have you exercised at least once a week?”), depression status (yes and no, in response to answering the question: “In the past year, have you experienced persistent depression for more than 2 weeks?” [23]), and number of chronic diseases based on the list recorded in the survey: hypertension, diabetes mellitus, cancer, lung disease, liver disease, heart disease, cerebrovascular disorders, psychiatric disorders, arthritis or rheumatism, prostate disease, diseases related to the digestive system, disk diagnosis, and dementia.

### Statistical Analyses

The data were analyzed by using R software version 4.1.3. Bivariate statistics was used to identify participant characteristics and estimate differences in ADL/IADL disability levels for research purposes. The prevalence of ADL/IADL disability levels was specified. The Chi-square test [24] was used to calculate *p*-values for differences across ADL/IADL disability levels.

Multinomial logistic regression models [25] were used to evaluate the factors predicting the presence of ADL/IADL disability. In the models, ADL and IADL disabilities were the dependent variables, and the other variable were predictors. The “none” category was designated as the reference category for both models, and the other categories were separately regressed against it. The jointly maximum likelihood method [26], an extension of the maximum likelihood method for a number of categories greater than two, was used to estimate the models. The Wald test [25] determined statistically significant differences in the estimates, and the Goodness-of-fit test [27] confirmed the suitability of the models. We reported the odds ratios (OR) obtained by exponentiation of the regression coefficients and the corresponding 95% confidence intervals (95% CI) for each model. ORs corresponding to *p*-values less than 0.05 in the two-sided Wald test were considered statistically significant. An OR represented a relative risk ratio for the change of the dependent variable in a particular category (relative to the reference category) associated with a one-level change in the respective predictor [25].

## Results

### Participant Characteristics

We analyzed 4,337 participants aged 65 and older, and 2,509 (57.85%) of them were female. People aged 65–74 accounted for the highest proportion (47.06%), whereas those aged 85 and older made up only 13.77% of the study population. People with an elementary school or lower education accounted for the highest proportion (approximately 50%), whereas those with a college degree or higher accounted for a relatively small proportion (8.72%). Up to 24.9% of participants reported being obese whereas only 3.76% of them were underweight. More than 60% of participants responded did not exercise at least once a week, and 4.45% of those living in a depressive state. Those with at least one chronic disease accounted for about 81%; three or more chronic diseases accounted for 26.59%. The proportion of people with at least one ADL limitation was 6.14%, of which severe disability was 1.94%. For the IADL limitation, these numbers were 15.49% and 3.11%, respectively. The demographic and health parameter characteristics of the participants are shown in Table 1.

**TABLE 1:** Characteristics of participants (the Korean Longitudinal Study of Aging Survey, South Korea, 2020).

Variable	Number of participants (n)	Percentage (%)
All participants	4,337	100.00
Sex		
Male	1,828	42.15
Female	2,509	57.85
Age in years		
65–74	2,041	47.06
75–84	1,699	39.17
85 or older	597	13.77
Living arrangement		
With relatives	2,942	67.83
Living alone	1,395	32.17
Educational status		
Elementary school or lower	2,154	49.67
Middle school	764	17.61
High school	1,041	24.00
College or higher	378	8.72
Body mass index		
Normal	1,813	41.80
Underweight	163	3.76
Overweight	1,281	29.54
Obesity	1,080	24.90
Physical activity		
Yes	1,734	39.98
No	2,603	60.02
Depression status		
Yes	193	4.45
No	4,144	95.55
Number of chronic diseases		
0	823	18.97
1	1,248	28.78
2	1,113	25.66
3 or more	1,153	26.59
ADL disability		
Severe	84	1.94
Moderate	182	4.20
None	4,071	93.86
IADL disability		
Severe	135	3.11
Moderate	537	12.38
None	3,665	84.51

ADL, activities of daily living; IADL, instrumental activities of daily living.

### Prevalence of ADL/IADL Disability Among Older Adults

The prevalence of ADL/IADL disability among older adults is shown in Table 2. The prevalence of severe ADL disability was significantly higher in females than in males (2.35% versus 1.37%). The same was true for severe IADL disability. However, while the prevalence of moderate ADL disability was higher in females than in males (4.31% versus 4.05%), moderate IADL disability was significantly lower in females than males (11.52% versus 13.57%). The prevalence of functional limitations increased with age. Specifically, the rates of severe ADL and severe IADL disabilities in people aged 65–74 were only 0.39% and 0.83%, respectively; in those aged 75–84, they were 1.88% and 2.71%; and in those aged 85 or older, they were 7.37% and 12.06%. The same was true for moderate ADL/IADL disability. The prevalence of severe and moderate ADL/IADL disability was significantly higher among people with an elementary or lower degree than in those with higher education levels. The prevalence of severe ADL disability in physically inactive people was 3.07%, much higher than in physically active people (0.23%). Those rates for severe IADL disability were 4.76% and 0.63%, respectively. For people without depression, the prevalence of severe disability was only 1.57% (ADL) and 2.65% (IADL). Meanwhile, it was 9.84% (ADL) and 12.95% (IADL) for people with depression. The prevalence of severe or moderate ADL/IADL disability increased rapidly with the number of chronic diseases presented, especially in those with three or more chronic diseases.

**TABLE 2:** Prevalence of functional limitations among older adults (the Korean Longitudinal Study of Aging Survey, South Korea, 2020).

Variable	ADL disability				IADL disability			
	Severe (%)	Moderate (%)	None (%)	*p*-value^a^	Severe (%)	Moderate (%)	None (%)	*p*-value^a^
Sex								
Male	1.37	4.05	94.58	0.060	2.68	13.57	83.75	0.058
Female	2.35	4.31	93.34		3.43	11.52	85.05	
Age in years								
65-74	0.39	1.52	98.09	<0.001	0.83	5.39	93.78	<0.001
75-84	1.88	3.89	94.23		2.71	13.71	83.58	
85 or older	7.37	14.24	78.39		12.06	32.50	55.44	
Living arrangement								
With relatives	1.43	2.89	95.68	<0.001	2.18	10.16	87.66	<0.001
Living alone	3.01	6.95	90.04		5.09	17.06	77.85	
Educational status								
Elementary school or lower	2.79	5.94	91.27	<0.001	4.22	16.30	79.48	<0.001
Middle school	1.05	2.88	96.07		1.96	9.17	88.87	
High school	0.96	2.50	96.54		2.02	8.26	89.72	
College or higher	1.59	1.59	96.82		2.12	7.94	89.94	
Body mass index								
Normal	2.48	4.52	93.00	<0.001	3.81	13.07	83.12	<0.001
Underweight	7.98	14.72	77.30		12.27	26.38	61.35	
Overweight	0.94	2.81	96.25		1.80	11.16	87.04	
Obesity	1.30	3.70	95.00		12.27	26.38	61.35	
Physical activity								
Yes	0.23	1.38	98.39	<0.001	0.63	7.44	91.93	<0.001
No	3.07	6.07	90.86		4.76	15.68	79.56	
Depression status								
Yes	9.84	12.44	77.72	<0.001	12.95	25.39	61.66	<0.001
No	1.57	3.81	94.62		2.65	11.78	85.57	
Number of chronic diseases								
0	0.36	1.58	98.06	<0.001	0.73	5.95	93.32	<0.001
1	1.13	2.48	96.39		1.68	9.14	89.18	
2	1.98	4.31	93.71		3.41	12.58	84.01	
3 or more	3.90	7.81	88.29		6.08	20.29	73.63	

^a^ Chi-squared test is used to calculate *p*-values for the differences across ADL/IADL disability levels.

ADL, activities of daily living; IADL, instrumental activities of daily living.

**FIGURE 2:**
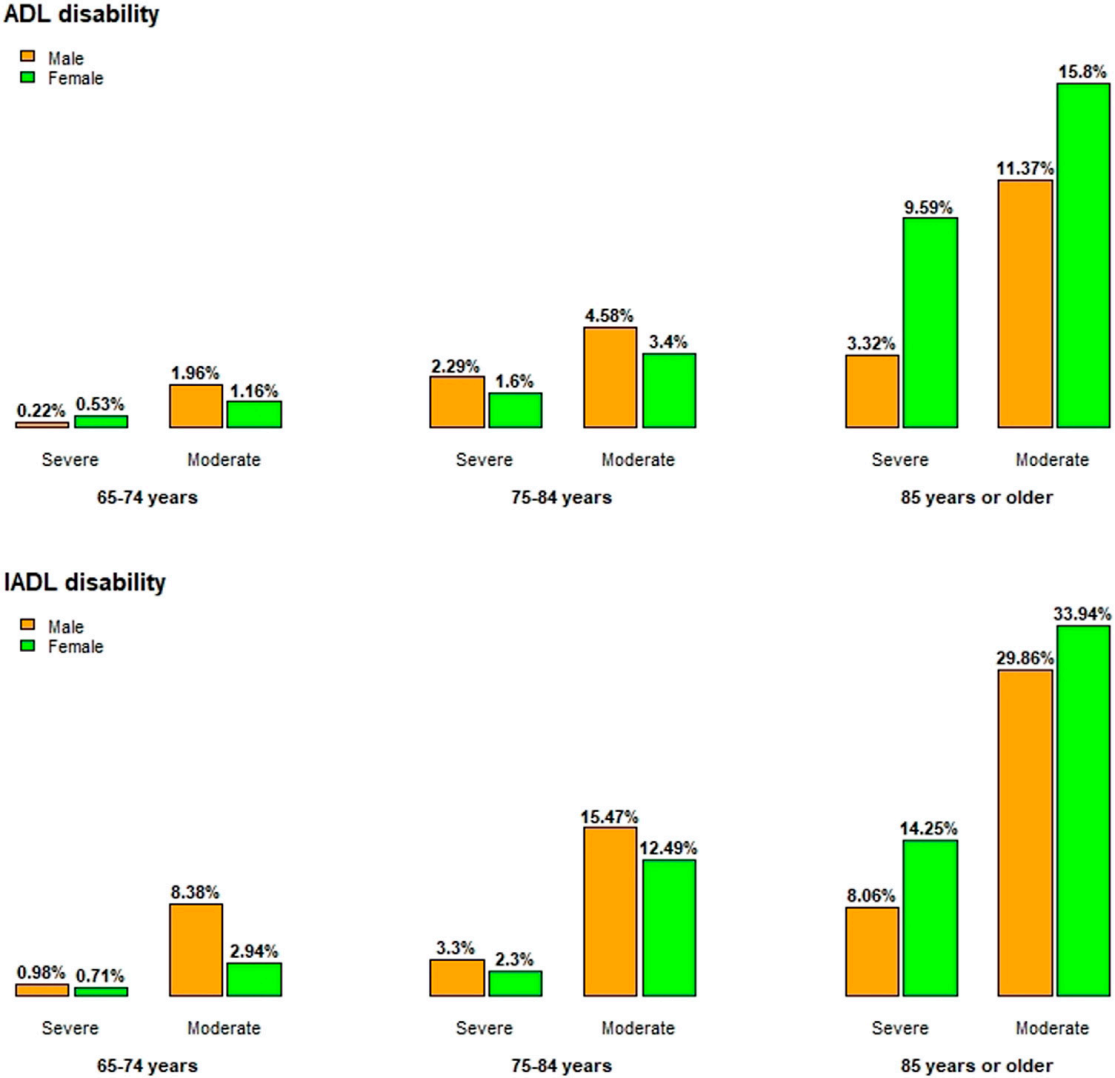
Prevalence of functional limitations by age and sex (the Korean Longitudinal Study of Aging Survey, South Korea, 2020).

The distributions of ADL/IADL disability levels by age and sex are visualized in Figure 2. As shown in the figure, ADL/IADL disability level increased rapidly with age and differed between males and females. Among people aged 84 or younger, the rate of ADL/IADL disability was higher in males than in females. On the contrary, among people aged 85 or older, the rate of ADL/IADL disability was significantly higher in females than in males.

### Multinomial Logistic Regression Analysis

The results of multinomial logistic regression analyses are shown in Table 3. The 5% level of significance verified the statistically significant attributes of the models. The extremely small *p*-values in the Goodness-of-fit test (around 2.2 × 10^−16^) indicated that the models were appropriate and consistent.

**TABLE 3:** Results of multinomial logistic regression analysis for functional limitations (the Korean Longitudinal Study of Aging Survey, South Korea, 2020).

Variable	ADL disability		IADL disability	
	Severe, OR (95% CI)^a^	Moderate, OR (95% CI)^a^	Severe, OR (95% CI)^a^	Moderate, OR (95% CI)^a^
Sex (Reference category: Male)				
Female	1.647*** (1.444; 1.879)	0.629** (0.579; 0.684)	0.867* (0.783; 0.963)	0.534*** (0.508; 0.562)
Age in years (Reference category: 65–74)				
75-84	3.377*** (2.841; 4.016)	1.753*** (1.589; 1.933)	2.435*** (2.147; 2.762)	2.112*** (1.947; 2.234)
85 or older	14.048*** (11.759; 16.782)	6.144*** (5.551; 6.802)	14.073*** (12.376; 16.003)	6.643*** (6.239; 7.074)
Living arrangement (Reference category: with relatives)				
Living alone	0.744** (0.661; 0.837)	1.508** (1.389; 1.636)	1.156* (1.051; 1.273)	1.288* (1.224; 1.355)
Educational status (Reference category: College or higher)				
Elementary school or lower	2.337*** (1.899; 2.876)	1.783*** (1.476; 2.154)	1.465** (1.226; 1.751)	1.448** (1.318; 1.591)
Middle school	2.536*** (1.982; 3.245)	1.626** (1.326; 1.993)	1.406* (1.149; 1.723)	1.198* (1.081; 1.328)
High school	2.176*** (1.725; 2.745)	1.508** (1.236; 1.843)	0.839 (0.694; 1.015)	1.098 (0.995; 1.213)
Body mass index (Reference category: Normal)				
Underweight	2.206*** (1.796; 2.666)	2.360*** (1.906; 2.844)	2.466*** (2.167; 2.806)	1.761*** (1.410; 2.126)
Overweight	0.422*** (0.366; 0.487)	0.715* (0.554; 0.982)	0.540*** (0.385; 0.702)	0.940 (0.794; 1.190)
Obesity	0.511*** (0.345; 0.686)	0.858 (0.686; 1.137)	0.547*** (0.390; 0.710)	0.840 (0.645; 1.038)
Physical activity (Reference category: Yes)				
No	9.846*** (7.898; 12.274)	3.232*** (2.934; 3.564)	6.096*** (5.313; 6.996)	1.887*** (1.798; 1.981)
Depression status (Reference category: No)				
Yes	5.558*** (4.903; 6.305)	2.904*** (2.611; 3.231)	5.039*** (4.501; 5.642)	2.413*** (2.227; 2.616)
Number of chronic diseases (Reference category: 0)				
1	2.648*** (2.011; 3.487)	1.357* (1.174; 1.569)	2.098*** (1.713; 2.569)	1.425** (1.318; 1.542)
2	4.165*** (3.188; 5.441)	2.146*** (1.868; 2.464)	4.084*** (3.367; 4.955)	1.842*** (1.705; 1.994)
3 or more	6.775*** (5.225; 8.786)	3.351*** (2.934; 3.827)	6.664*** (5.521; 8.045)	2.925*** (2.715; 3.151)

^a^ The odds ratio and corresponding 95% confidence intervals are calculated against no disability as the reference.

‘***’ *p* < 0.001, ‘**’ *p* < 0.01, ‘*’ *p* < 0.05, ‘’ *p* ≥ 0.05.

ADL, activities of daily living; IADL, instrumental activities of daily living.

As shown in Table 3, demographic and health parameters predicted the presence of ADL/IADL disability. Remarkably, factors strongly associated with ADL/IADL disability could be seen, such as sex, age, educational status, BMI, physical activity, depression status, and the number of chronic diseases. The relative risk for severe disability compared with no disability was 3.38 times (ADL) and 2.44 times (IADL) higher when moving from ages 65–74 to 75–84 years, and it increased by 14.05 times (ADL) and 14.07 times (IADL) when moving to ages 85 years or older. These numbers were much stronger than the relative risk for moderate disability compared with no disability: 1.75 times and 6.14 times higher (ADL); 2.11 times and 6.64 times higher (IADL), respectively. In terms of educational status, the relative risk for severe ADL disability compared with no ADL disability was 2.34 times higher when moving from college or higher to elementary school or lower, 2.54 times higher when moving to middle school, and 2.18 times higher when moving to high school. The relative risk for ADL/IADL disability compared with no ADL/IADL disability were 2.21 and 2.47 times higher when moving from a normal weight to underweight, respectively. The severity of ADL/IADL disability also increased significantly in physically inactive versus physically active individuals and in depressed versus non-depressed individuals. The relative risk for severe disability compared with no disability was 9.85 times (ADL) and 6.10 times (IADL) higher when moving from physically active to inactive. Similarly, the relative risk was 5.56 times and 5.04 times higher, respectively, when moving from a non-depressed to a depressed state. Furthermore, the severity of ADI/IADL disability increased rapidly with number of chronic diseases. The relative risk for severe disability compared with no disability was 2.65 times (ADL) and 2.10 times (IADL) higher when moving from a person without chronic diseases to a person with only one chronic disease, and it reached 6.78 times and 6.66 times higher, respectively, when moving to a person with three or more chronic diseases.

## Discussion

People with severe functional limitations often experience particular difficulties with daily activities, which place a burden on the healthcare system [14–16]. Therefore, before making a healthcare plan, it was essential to identify predictive factors that were significantly related to functional limitations in the performance of basic and complex activities of daily living (ADLs and IADLs) [4, 9, 10]. This study developed a model to verify the relationships between demographic and health parameters and functional limitations in daily living, defined as ADL/IADL disability levels, among older adults in Korea. This study used data from the KLOSA survey in 2020.

This study analyzed the prevalence of functional limitations in older Korean adults. For the entire population discussed in this study, the proportion of people who reported at least one ADL limitation was 6.14%, of which severe ADL disability was 1.94%. These numbers for IADL disability were 15.49% and 3.11%, respectively. The ADL/IADL disability increased significantly with age. For people aged 85 years or older, approximately 21.6% reported at least one ADL limitation, and 44.6% reported at least one IADL limitation. The results are similar to those of a survey of older adults residing in the community in Korea [8]. Overall those rates in Korean older adults were significantly lower than those observed in recent studies: in a study in southeastern Poland, approximately 17.1% of older adults had at least one ADL limitation, and 35.8% of them had at least one IADL limitation [7]; those rates were 22% and 48%, respectively in India [6]. In another study using the Survey of Health, Ageing and Retirement in Europe, data from 17 countries reported that ADL and IADL limitations were 11.7% and 17.6%, respectively, and the incidence of ADL/IADL disability by region was as follows: Northern Europe (5.6%/13.8%), Western Europe (11.0%/16.3%), Southern Europe (12.0%/18.9%), and Eastern Europe (14.0%/20.6%) [28]. Although it is difficult to compare directly due to the difference in the age of the participants and the scale of determining the disability of each country, it can be seen that the incidence of disability in older adults in Korea is relatively low, considering that most of the studies were conducted on people aged 50 years and older. As mentioned in the study of Scheel-Hincke [28], the disability level was higher in countries with less developed social policies and higher socioeconomic inequality. Our findings were important for formulating social policies and improving the Korean healthcare system to properly accommodate the development of physical disabilities among older people.

The average human lifespan in Korea has been increasing in recent years, and the number of older adults increased significantly. Around 16% of the Korean population was age 65 or older in 2020, according to the South Korea Age structure [29]. Among them, approximately 31% of the people in this study lived alone, and most of them (85.75%) were female. According to other national data, 19.8% (27.4% of females and 9.7% of males) of older adults lived alone [8]. In this study, 13.8% of the population was aged 85 or older, which was a higher proportion than the 6.4% in the other national survey, indicating that the number of older adults living alone increased with age. Considering the high proportion of females living alone, it was necessary to prepare a healthcare policy to prevent the progression of disability in this vulnerable group.

In this study, we verified that advanced age, low education, being underweight, physical inactivity, and chronic diseases were factors strongly associated with functional limitations in older adults. These findings were consistent with those of previous studies [6, 7, 11]. In this study, those with an elementary or lower education were 2.34 times more likely to have severe ADL disability than those with a college/university education. Age was identified as the most significant risk factor for ADL/IADL disability. Especially, as shown in the study, ADL/IADL increased rapidly with advanced age, similar to the results in previous studies [6, 8, 30]. However, by considering both sex and age, the prevalence rates of ADL/IADL disability were higher in males aged 84 years or younger and in females aged 85 years or older. This finding differed from previous studies, in that ADL/IADL disability was higher in females than in males in all age groups [8, 30]. It was necessary to further examine the factors that influence functional disability in males aged 65–84 years. Furthermore, an increasing trend of the global older population can be predicted to increase the number of older adults with ADL/IADL disabilities. A recent systematic review study reported that disability can be improved through individually customized interventions [31], especially focusing on the modifiable factors of ADL/IADL disability, such as physical activity, depression, and abnormal BMI.

We found that the prevalence of ADL/IADL disability was higher in underweight individuals than in normal-weight individuals, while it was lower in groups with overweight and obesity. This finding differs from the studies from Korea in that BMI did not significantly affect the prevalence of ADL/IADL disability in Korean older adults [18, 19]. Previous studies cannot be directly compared with this study due to differences in participants’ age and data collection period. In particular, these studies [18, 19] used the publicly available data collected 6–14 years before this present study. A great difference in the distribution of BMI category was observed; the prevalence of the obesity group was 24.9% in this study, and 4.2% in the previous study [19]. Because the results of this study may reflect the changes in dietary habits in older adults in Korea, further study should be interesting in identifying the effect of BMI on ADL/IADL disability in this population. Although the results regarding the relationship between BMI and ADL/IADL disability are inconsistent, BMI is still important in the older population. A recent study found a u-shaped relationship between functional disability and BMI in older adults [32]. That was, both underweight and obesity, including abdominal obesity, were risk factors for ADL disability, whereas overweight tended to correlate with decreased ADL disability. In the older population, malnutrition due to poor protein intake and decreased mobility due to obesity or abdominal obesity were closely related to sarcopenia [12], and an increase in sarcopenia was closely related to an increase in both ADL disability and frailty [33], so an interventional approach to maintaining proper nutritional status was needed.

The rates of severe ADL/IADL disability were exceptionally high in physically inactive individuals (a 9.85-fold increase for ADL and a 6.10-fold increase for IADL as shown in this study) compared with physically active individuals. A previous study reported a high prevalence of ADL (OR = 15.23) and IADL (OR = 4.98) disability in older adults without physical activity [7]. Limitations in physical activity led to physical and mental disorders and affected the ability to maintain independence in daily living [34]. However, older adults can obtain health benefits when they participate in physical activity for a sufficient period [35]. Policy support at the local and national levels is needed for the older population to slow or improve the progression of disability through physical activities. The same is true for people with depression or those with at least one chronic disease. Previous studies reported depression as a major factor in ADL/IADL disability [20, 36]. Depressive symptoms in older adults were also associated with the occurrence of ADL/IADL disability after 2 years [37]. Furthermore, because depression can be both a cause and a consequence of ADL/IADL disability [37], it was essential to verify the depression status of older adults before determining the progression of their healthcare.

Chronic disease has been consistently reported as a risk factor for disability, and it may lead to disability due to limitations on physical and social activities [18, 38]. The high prevalence of chronic disease in older adults in Korea [8] led to an increase in disability, which can be a social burden. However, if chronic diseases are properly managed, it would be possible to block the progression to disability by providing a program for chronic disease management and prevention of aggravation. This study also found that the risk of ADL/IADL disability was higher among people living alone than in those living with relatives. Receiving the support of relatives reduced the stress caused by chronic diseases and functional decline in older adults [7, 16].

We also found that factors such as age, physical activity, depression, and chronic diseases influenced the risk for severe disability much more strongly than that for moderate disability at both ADL and IADL while there was almost no more difference in the influence of BMI on risk for severe as well as moderate disability. With the factor of educational status, the risk of ADL disability was significantly higher than that of IADL disability at both severe and moderate levels. This finding is interesting and has not been shown in previous studies related to older adults in Korea. This can be explained by the nature of limitations. ADL disability involves basic activities of daily living while the IADL disability includes more complex activities, so the prevalence of IADL disability was often higher than that of ADL disability and it is often more evident in the later stages of the disability [39].

This study had some limitations. First, several parameters, such as the place of residence and income, were not included in this study to avoid the presence of missing data. Second, as this was a secondary data analysis study, we were unable to include the variables known as significantly associated factors (e.g., frailty, walking ability, number of medications, quality of life) as in previous studies. Third, our study was based on cross-sectional data and did not include time-varying trends in ADL/IADL disability. It is necessary to confirm the causal relationship between the associated factors according to the level of ADL/IADL disability in a future longitudinal study. Despite several limitations, our findings have importance in formulating social policies and improving the healthcare system to properly accommodate the development of physical disabilities among older people in Korea. In particular, active policy support and intervention approaches for severe disability groups will be needed.

In summary, this study specified the risk factors associated with ADL/IADL disability among older adults in Korea using bivariate analyses and multinomial logistic regression analyses of KLOSA data. Significantly, severe ADL/IADL disability tended to be closely associated with age, educational status, BMI, physical activity, depression status, and chronic diseases. In particular, modifiable factors were identified as major related factors of ADL/IADL disability, and it is necessary to prevent the occurrence of disability in the older population by improving modifiable factors through active intervention.
